# Superior persistence of ustekinumab compared to anti-TNF in vedolizumab-experienced inflammatory bowel diseases patients: a real-world cohort study

**DOI:** 10.1186/s12876-024-03577-1

**Published:** 2024-12-31

**Authors:** Horng-Yih Chiu, Chia-Jung Kuo, Ming-Wei Lai, Ren-Chin Wu, Chien-Ming Chen, Cheng-Tang Chiu, Yu-Bin Pan, Cheng-Hsun Chiu, Puo-Hsien Le

**Affiliations:** 1 School of Medicine, Chang Gung University, Taoyuan City, Taiwan; 2 Department of Gastroenterology and Hepatology, Linkou Branch, Chang Gung Memorial Hospital, 5, Fu-Hsin Street, Guei-Shan District, Taoyuan, 33305 Taiwan; 3 Inflammatory Bowel Disease Center, Chang Gung Memorial Hospital, Taoyuan, Taiwan; 4 Chang Gung Microbiota Therapy Center, Chang Gung Memorial Hospital, Taoyuan, Taiwan; 5 Taiwan Association for the Study of Small Intestinal Diseases (TASSID), Taoyuan, Taiwan; 6 Division of Pediatric Gastroenterology, Department of Pediatrics, Linkou Branch, Chang Gung Memorial Hospital, Taoyuan, Taiwan; 7 Department of Anatomic Pathology, Linkou Branch, Chang Gung Memorial Hospital, Taoyuan, Taiwan; 8 Department of Medical Imaging and Interventions, Linkou Branch, Chang Gung Memorial Hospital, Taoyuan, Taiwan; 9 Biostatistics Unit, Clinical Trial Center, Linkou Branch, Chang Gung Memorial Hospital, Taoyuan, Taiwan; 10 Division of Pediatric Infectious Diseases, Department of Pediatrics, Linkou Branch, Chang Gung Memorial Hospital, Taoyuan, Taiwan

**Keywords:** Inflammatory bowel disease, Vedolizumab, Ustekinumab, Anti-TNF, Persistence

## Abstract

**Background/Aims:**

The increasing use of biologic therapies for moderate to severe inflammatory bowel disease (IBD) highlights the importance of optimal treatment sequencing, particularly after vedolizumab (VDZ) exposure. Studies comparing the effectiveness of ustekinumab (UST) and antitumor necrosis factor (anti-TNF) agents post-VDZ are limited.

**Methods:**

This retrospective study analyzed VDZ-experienced IBD patients treated with UST or anti-TNF (adalimumab and infliximab) from May 2019 to January 2024. We conducted a comparative analysis of the 52-week treatment persistence between UST and anti-TNF therapies, while also identifying independent predictors that influence 52-week persistence.

**Results:**

The study included 110 participants, with 40 diagnosed with ulcerative colitis (UC) and 70 with Crohn’s disease (CD). Demographics were comparable across treatment groups. The primary discontinuation reason for VDZ was secondary non-response. Kaplan-Meier analysis revealed that UST demonstrated superior 52-week persistence in overall IBD, CD and UC patients, compared to anti-TNF. Cox regression analysis also showed UST’s superiority in overall IBD (HR: 0.15, 95% CI: 0.05–0.45, *p* < 0.001), CD (HR: 0.09, 95% CI: 0.01–0.68, *p* = 0.02), and UC (HR: 0.28, 95% CI: 0.08–0.996, *p* = 0.049). The independent predictors for 52-week treatment persistence are Crohn’s disease (Odds Ratio: 7.151, 95% CI: 1.763–28.995, *p* = 0.006) and UST treatment (Odds Ratio: 7.912, 95% CI: 1.789–34.992, *p* = 0.006). Notably, UST required more frequent dosing adjustments than anti-TNF, although both treatments exhibited comparable safety profiles.

**Conclusions:**

UST demonstrated superior 52-week treatment persistence in IBD patients previously treated with VDZ compared to anti-TNF agents, albeit with a need for more frequent dose adjustments.

## Background

Inflammatory bowel disease (IBD), comprising Crohn’s disease (CD) and Ulcerative colitis (UC), manifests as chronic, relapsing inflammation in the gastrointestinal tract, often resulting in significant intestinal damage and disability [1]. Biologic therapies have notably reduced the necessity for colectomy and enhanced clinical outcomes [2, 3]. The Selecting Therapeutic Targets in Inflammatory Bowel Disease (STRIDE-II) initiative highlights the critical importance of achieving endoscopic remission, linking it to improved long-term outcomes and potential reduction in bowel damage [4, 5]. Additionally, transmural healing in CD and histological healing in UC are emerging as pivotal adjunct targets, potentially offering better prediction of long-term remission and aiding in the prevention of colitis-associated cancer [4, 6]. In this evolving landscape, the sustained effectiveness of advanced therapies is paramount, as it mirrors their efficacy, the potential for anti-drug antibody development, and overall safety in practical clinical settings.

Vedolizumab (VDZ), a gut-specific monoclonal antibody targeting α4β7 integrin, stands out for its excellent safety profile and is recommended as a first-line treatment for moderate to severe IBD [7–10]. Notably, in adult outpatients with moderate to severe UC, especially those naive to biologic agents, the American Gastroenterological Association (AGA) endorses using infliximab or vedolizumab over adalimumab for inducing remission, a recommendation influenced by the findings of the VARSITY study [11, 12]. However, the current literature is limited in this area, with only a single study involving fifty-nine CD patients suggesting comparable efficacy between UST and anti-TNF therapies following treatment with VDZ [13]. There remains a notable absence of comprehensive research comparing the efficacy and safety across the full spectrum of IBD, encompassing both CD and UC patients. Such comprehensive analysis is crucial for making well-informed therapeutic decisions. Our study, therefore, aims to bridge this gap by comparing the persistence and safety profiles of anti-TNF and UST in a broad cohort of IBD patients who have previously received VDZ treatment. This research endeavor is poised to offer significant insights, contributing to the optimization of treatment strategies in this patient population.

## Methods

This study was a single-center retrospective cohort analysis conducted at Chang Hung Memorial Hospital at Linkou, one of Taiwan’s largest tertiary medical centers with 4000 beds. We enrolled IBD patients who had previously been treated with VDZ. These patients were categorized into anti-TNF and UST groups based on their subsequent biologic treatment from May 2019 to January 2024. The anti-TNF medications included adalimumab (ADA) and infliximab (IFX), no other anti-TNF agents were used. We extracted clinical data from electronic medical records, gathering information such as age, gender, body mass index (BMI), diagnosis (CD/UC), reasons for discontinuing VDZ and the post-VDZ biologics, therapeutic duration, number of IBD-related hospitalizations, and incidence of opportunistic infections like CMV colitis, *Clostredioides difficile* and *Clostridium innnocuum* infections. No patients were excluded.

In Taiwan, biologics usage is under strict criteria set by National Health Insurance program (NHI). First, the patients should have registered for catastrophic illness and had undergone conventional therapy (including 5-ASA, steroid and immunosuppressive agents) for a minimum of 6 months. The term “catastrophic illness” is defined by Taiwan’s NHI program as a designation for conditions requiring chronic treatment and significantly impacting daily life. Patients certified under this category are eligible for partial medical expense exemptions. In Taiwan, Crohn’s disease and ulcerative colitis are recognized as catastrophic illnesses under this program. Secondly, if conventional therapy failed or lead to adverse events, the disease severity must meet specific criteria: a CDAI score of ≥ 300 or a CDAI score of ≥ 100 with prior CD-related surgery for CD patients, and a Mayo score of ≥ 9 with a Mayo endoscopy subscore of ≥ 2 for UC patients. Also, reimbursement provided only 12 months of treatment period. Following the 12-month treatment period, patients must either transition to self-funded treatment or discontinue treatment for a mandatory drug holiday of three months. During this hiatus, they can reapply for reimbursement once their disease severity meets the previously specified criteria [14]. 

In this study, primary non-response was categorized as treatment failure during the induction phase, while secondary non-response was identified during the maintenance phase. The standard dosing intervals for the treatments varied: VDZ was administered at a dosage of 300 mg initially at weeks 0, 2, and 6, followed by a maintenance dose every 8 weeks. ADA dosing commenced with 160 mg at week 0, 80 mg at week 2, 40 mg at week 4, and subsequently 40 mg biweekly. IFX involved a dose of 5 mg/kg at weeks 0, 2, and 6, then continued every 8 weeks. An increase in the IFX dose to 10 mg/kg was not permitted in this study. For UST, the treatment regimen included an initial dose of 260 mg for patients weighing ≤ 55 kg, 390 mg for those between 55 and 85 kg, and 520 mg for patients weighing ≥ 85 kg at week 0, followed by 90 mg at week 8, and thereafter a regular administration every 12 weeks. Dose escalation was defined as a shorter interval than standard.

Patient demographics and clinical data were presented as mean ± standard deviation or proportions. We used t-tests for continuous data and Chi-square tests for categorical data. Persistence was measured as continued medication use, while non-persistence was defined as failing to refill a prescription within a set period. Kaplan-Meier (KM) survival curves illustrated persistence over time, with statistical differences assessed using the log-rank test. Cox proportional hazards models were employed to assess the 52-week treatment retention comparing UST and anti-TNF therapies. Both univariate and multivariate logistic regression analyses were utilized to identify predictors of 52-week treatment persistence. In multivariable analysis, stepwise regression was conducted. A *p*-value < 0.05 was considered statistically significant. Univariate and multivariate analysis was conducted on SPSS version 24.0. Other analyses were conducted using R software version 4.1.3.

## Results

Our study included 110 IBD patients with prior VDZ treatment, who received either anti-TNF or UST therapy from May 2019 to January 2024. Baseline characteristics including age, gender, and BMI were comparable across both treatment groups (Table 1). Secondary non-response was the primary reason for discontinuing VDZ and also led to the discontinuation of the post-VDZ biologic therapy (anti-TNF/UST).

**Table 1:** Baseline characteristics of vedolizumab-experienced inflammatory bowel disease patients

Characteristics	Overall (*n* = 110)	Anti-TNF (*n* = 51)	UST (*n* = 59)	*P*
Age	47.5 ± 17.1	47.8 ± 17.0	47.2 ± 17.3	0.846
Gender (Male)	75 (68.2)	37 (72.5)	38 (64.4)	0.361
BMI	22.8 ± 7.1	24.3 ± 9.6	21.4 ± 3.2	0.059
IBD types				
Ulcerative colitis	40 (36.4)	24 (47.1)	16 (27.1)	0.030^*^
Crohn’s disease	70 (63.6)	27 (52.9)	43 (72.9)	
Dose escalation	17 (15.5)	4 (7.8)	13 (22.0)	0.040^*^
Concurrent thiopurine use	28 (25.5)	13 (25.5)	15 (25.4)	0.994
Steroid free at 52 weeks	77.5	73.7	81.0	0.583
Reasons of VDZ discontinuation				
Reimbursement restriction (52 weeks)	26 (23.6)	7 (13.7)	19 (32.2)	0.023^*^
Side effects	2 (1.8)	2 (3.9)	0	0.125
Primary non-response	24 (21.8)	14 (27.5)	10 (16.9)	0.184
Secondary loss of response	58 (52.7)	28 (54.9)	30 (50.8)	0.671
Reasons for anti-TNF/UST discontinuation				
Side effects	1 (4)	1 (4.8)	0	0.656
Primary non-response	1 (4)	1 (4.8)	0	0.656
Secondary loss of response	19 (76)	15 (71.4)	4 (100)	0.220
Personal reasons	1 (4)	1 (4.8) ^a^	0	0.656
Others	3 (12)	3 (14.3) ^b^	0	0.420
IBD-related hospitalization	21 (19.1)	14 (31.8)	7 (17.1)	0.115
Opportunistic infections				
CMV colitis	3 (2.7)	3 (6.8)	0	0.089
Average time to first infection (weeks)	21.1	21.1	-	-
*C. difficile* infection	7 (6.4)	5 (11.4)	2 (4.9)	0.277
Average time to first infection (weeks)	18	20	13	0.571
*C. innocuum* infection	9 (8.2)	7 (15.9)	2 (4.9)	0.099
Average time to first infection (weeks)	21.8	19.8	29.1	0.667

Continuous data in the study were analyzed using the t-test and are presented as mean ± standard deviation. Categorical data were evaluated using Pearson’s Chi-Square test and are displayed as absolute numbers (percentage). Abbreviations: Anti-TNF, anti- tumor necrosis factor; AZA, azathioprine ; BMI, body mass index; *C. difficile* infection, *Clostredioides difficile* infection; *C. innocuum*, *Clostridium innocuum*; CMV, cytomegalovirus; IBD, inflammatory bowel disease; UST, ustekinumab; VDZ, vedolizumab. *P < 0.05. ^a^. The patient refused further treatment due to the long infusion time required for infliximab

^b^. One adalimumab user discontinued treatment because of the COVID-19 pandemic and was unable to return for further treatment; two infliximab users discontinued treatment due to death from intracranial hemorrhage, and another patient received dual biological treatment with ustekinumab and infliximab, then continued with ustekinumab under stable condition

**Fig. 1:**
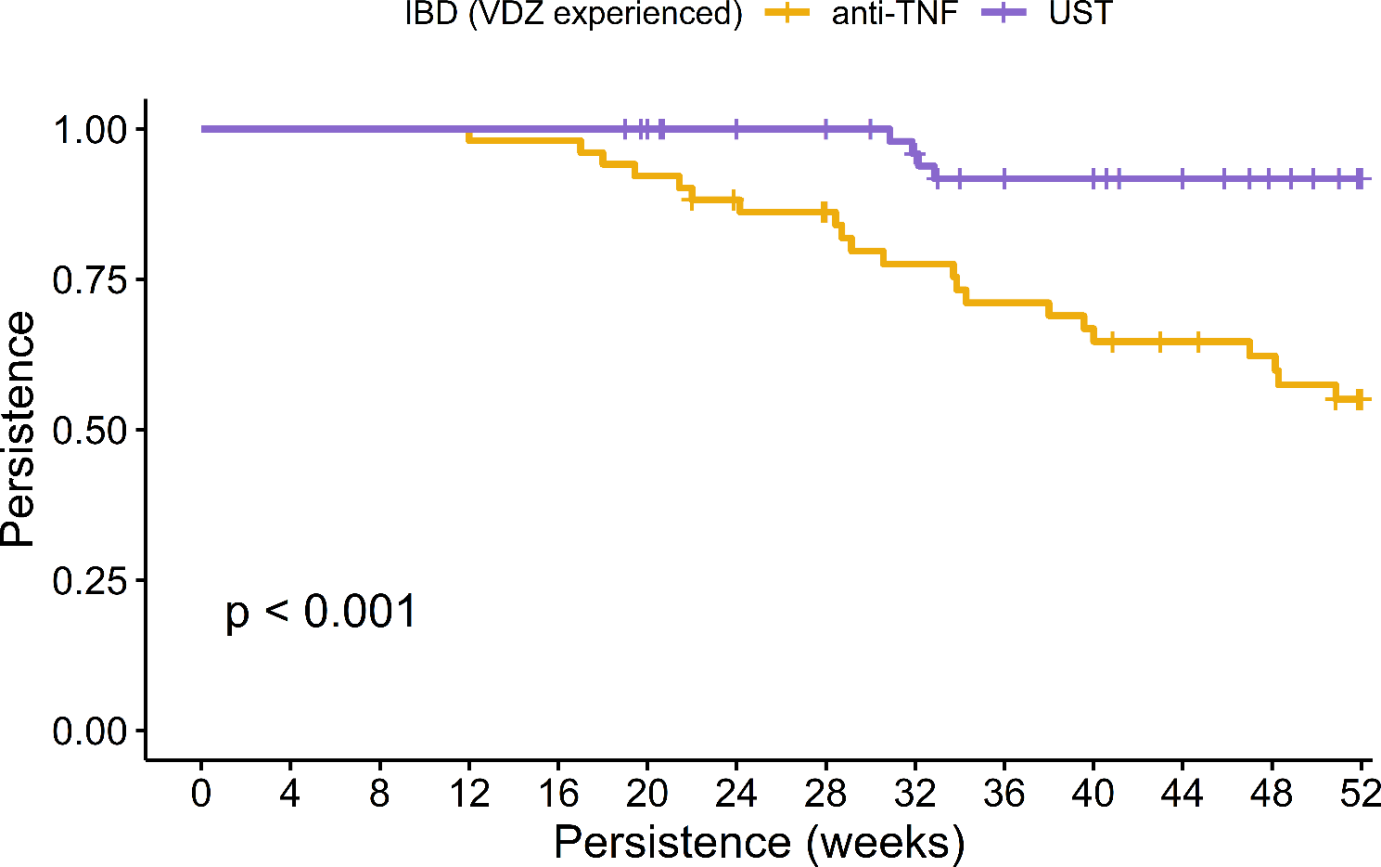
Comparative Analysis of 52-Week Treatment Persistence Between Anti-TNF and Ustekinumab in Vedolizumab-Experienced Inflammatory Bowel Disease Patients

**Fig. 2:**
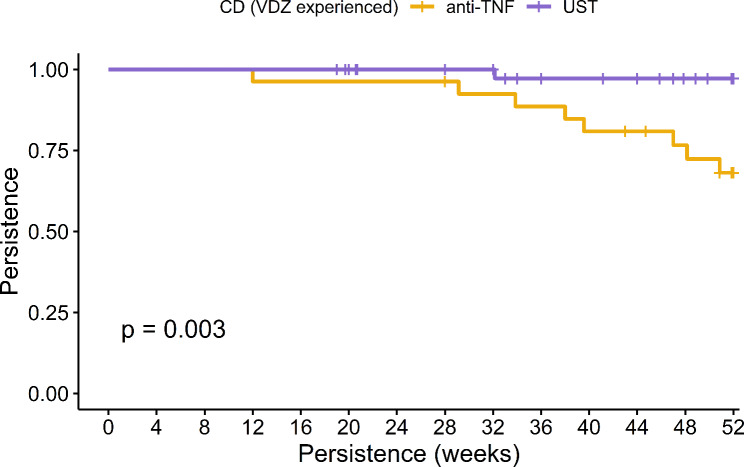
Comparative Analysis of 52-Week Treatment Persistence Between Anti-TNF and Ustekinumab in Vedolizumab-Experienced Crohn’s Disease Patients

**Fig. 3:**
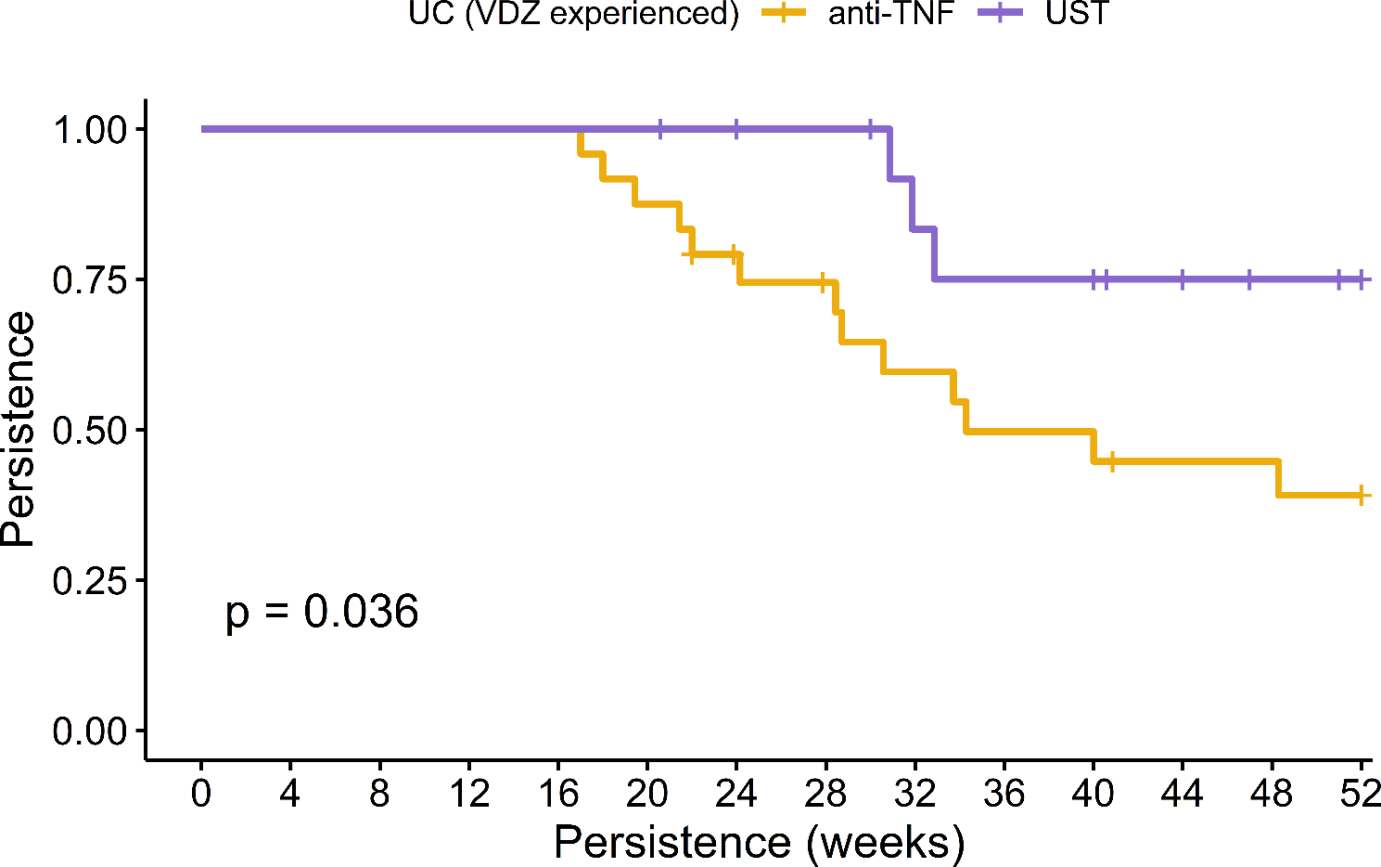
Comparative Analysis of 52-Week Treatment Persistence Between Anti-TNF and Ustekinumab in Vedolizumab-Experienced Ulcerative Colitis Patients

In the overall cohort of IBD patients, the 52-week persistence rates were observed to be 52.27% for anti-TNF therapy and 91.67% UST. Notably, UST exhibited significantly enhanced persistence as evidenced in both KM analysis (log-rank *p* < 0.001, as shown in Fig. 1) and Cox regression analysis (HR: 0.15, 95% CI: 0.05–0.45, *p* < 0.001). Specifically in CD patients, these rates were 66.67% for anti-TNF and an impressive 97.22% for UST, with UST demonstrating superior persistence in both KM analysis (log-rank *p* = 0.003, Fig. 2) and Cox regression analysis (HR: 0.09, 95% CI: 0.01–0.68, *p* = 0.02). Among ulcerative colitis (UC) patients, the persistence rates were 35% for anti-TNF and 75% for UST, with UST again showing greater persistence as indicated by KM analysis (log-rank *p* = 0.036, Fig. 3) and Cox regression (HR: 0.28, 95% CI: 0.08–0.996, *p* = 0.049). Furthermore, logistic regression analysis identified Crohn’s disease (Odds Ratio: 7.151, 95% CI: 1.763–28.995, *p* = 0.006) and UST treatment (Odds Ratio: 7.912, 95% CI: 1.789–34.992, *p* = 0.006) as the independent predictors for 52-week treatment persistence, as detailed in Table 2.

**Table 2:** Analysis of clinical factors associated with 52-week treatment persistence

Characteristics	Univariable			Multiple		
	OR	95% CI	*p*-value	OR	95% CI	*p*-value
Age	0.99	0.964–1.016	0.431	0.98	0.941–1.019	0.309
Male	1.011	0.388–2.631	0.982	1.010	0.245–4.160	0.989
BMI	1.012	0.962–1.065	0.64	1.043	0.980–1.109	0.187
IBD types						
Ulcerative colitis	0.221	0.086–0.568	< 0.002*			
Crohn’s disease	4.519	1.759–11.606	< 0.002*	7.151	1.763–28.995	0.006*
Dose escalation	1.97	0.53–7.329	0.312	2.092	0.369–11.865	0.405
Reasons of VDZ discontinuation						
Reimbursement restriction (52 weeks)	2.72	0.743–9.959	0.131	-	-	-
Side effects	0	0	0.999	-	-	-
Primary non-responder	1.615	0.496–5.262	0.426	-	-	-
Secondary non-responder	0.549	0.219–1.38	0.202	-	-	-
Post-VDZ biologics						
Anti-TNF	0.104	0.033–0.331	< 0.001*			
UST	9.625	3.023–30.645	< 0.001*	7.912	1.789–34.992	0.006*
IBD related hospitalization	0.292	0.105–0.810	0.018*	0.285	0.047–1.727	0.172
CMV colitis	0.137	0.012–1.578	0.111	0.850	0.032–22.498	0.923
C. difficile colitis	0.719	0.131–3.951	0.704	7.970	0.677–93.804	0.099
C. innocuum infection	0.198	0.049–0.803	0.023*	0.420	0.047–1.727	0.436

Continuous data are presented as mean ± standard deviation. Categorical data are displayed as absolute numbers (percentage). Both were evaluated by univariate and multivariate logistic regression and the result are displayed. Abbreviations: Anti-TNF, anti- tumor necrosis factor; BMI, body mass index; *C. difficile* infection, *Clostrideoides difficile* infection; *C. innocuum*,* Clostridium innocuum*; CMV, cytomegalovirus; IBD, inflammatory bowel disease, OR, Odds Ratio; CI, confidence interval; UC, ulcerative colitis; UST, ustekinumab; VDZ, vedolizumab. *P < 0.05

During the 52-week treatment period, dose escalation was higher in the UST group (22.0%) than in the anti-TNF group (7.8%). The patients who underwent dose escalation had their treatment intervals shortened: for IFX, from every 8 weeks to every 4 weeks; for ADA, from every 2 weeks to every week; and for UST, from every 12 weeks to every 8 weeks. At 52 weeks, 73.7% of anti-TNF and 81% of UST patients were steroid-free. Safety profiles, including side effects, hospitalization, and opportunistic infections, showed no significant differences (Table 1).

## Discussion

There are more and more biologics and small molecule therapies available for IBD treatment, clinicians face uncertainties regarding patient responses to new advanced therapies. While research into predictive biomarkers and fecal microbiota signatures is inching us closer to precision medicine, their clinical application remains a distant goal [15, 16]. Additionally, a perceived ‘glass ceiling’ in achieving clinical and endoscopic remissions limits progress toward maximizing long-term health care-related quality of life for all IBD patients [17].

Selecting appropriate biologics for both naïve and experienced IBD patients is a critical issue. There are limited head-to-head studies to guide choices between biologics in naïve patients. The VARSITY trial highlighted VDZ’s superiority over ADA in UC, while the SEAVUE trial showed comparable efficacy between UST and ADA in CD [12, 18]. Emerging studies are now focusing on other advanced therapies and anti-TNF-experienced patients. Currently, efficacy and safety comparisons are largely drawn from systematic reviews, network meta-analyses, and real-world studies [19–21].

Anti-TNF, the first class of biologics used in IBD treatment, has been extensively studied, particularly regarding the choice of advanced therapies in anti-TNF-experienced patients [22–26]. Interestingly, in patients with prior failure to anti-TNF treatment, multiple studies also suggested that UST demonstrate superior persistence and treatment efficacy compared to VDZ [27–29]. VDZ, suitable for both biologic-naïve and experienced patients, is notable for its outstanding safety profile due to its gut-selective mechanism. A small study involving 27 anti-TNF experienced, VDZ failure CD patients reported a 55.5% steroid-free clinical remission rate with UST after 12 months [30]. Furthermore, one poster indicated that VDZ failure as a first-line biologic does not diminish the response rates to second-line therapy [31]. Another poster highlighted the efficacy and safety of anti-TNF post primary VDZ failure in 21 pediatric IBD patients [32]. To our knowledge, only one study with 59 CD patients has compared the effectiveness of anti-TNF or UST as a second-line biologic following VDZ failure, finding no significant differences in effectiveness between the two biologics [13].

Our research underscores crucial insights into the persistence and effectiveness of biologic treatments in IBD patients who were previously administered VDZ. The results reveal a significantly greater persistence rate for UST over anti-TNF therapies in both CD and UC cohorts. Furthermore, UST emerged as the sole independent factor predicting 52-week treatment persistence in IBD patients. This higher persistence with UST might be attributed to its high efficacy, low incidence of anti-drug antibodies, and favorable safety profile.

Ustekinumab, a fully-humanized immunoglobulin G monoclonal antibody targeting the p40 subunit common to both interleukin-12 and interleukin-23, has been approved for the treatment of moderate to severe CD and UC. In the United States, the standard UST dosing regimen for CD involves an intravenous induction dose of 6 mg/kg, followed by subcutaneous maintenance therapy administered every 8 weeks. European guidelines provide options for subcutaneous maintenance dosing either every 8 weeks or every 12 weeks. In Taiwan, the reimbursed maintenance dosing is scheduled every 12 weeks subcutaneously. However, researches have shown that an every-8-weeks dosing schedule increases the response and remission rates in both biologic-experienced CD and UC patients [33–36]. Furthermore, even with an 8-week maintenance dosing schedule, dose escalations were necessary for over 40% of UC patients and approximately 50% of CD patients [37–40]. In practical clinical scenarios, dose escalation of UST was effective in inducing a clinical response and attaining clinical remission in roughly 40% of IBD patients who exhibited an inadequate response or lost response to standard dose therapy [41, 42]. In our study, all patients previously received VDZ treatment. We observed that during the 52-week treatment period, the UST group had a significantly higher rate of dose escalation compared to the anti-TNF group (22% vs. 7.8%). This suggests a greater necessity for maintaining UST dosing every 8 weeks in biologic-experienced patients. Additionally, it indicates an increased need for intensified treatment to achieve or sustain disease control in patients who are transitioning to UST therapy. Despite this, a majority of patients in both treatment groups were steroid-free at 52 weeks, underscoring the efficacy of both therapeutic strategies in maintaining a steroid-free state.

It is important to note that, despite variations in treatment persistence and dose escalation, both groups showed no significant differences in their safety profiles. This includes aspects such as side effects, hospitalization rates, and the incidence of opportunistic infections. This observation underscores the exceptional safety profile of both biologic treatments. In summary, our findings underscore UST as a potentially more favorable biologic treatment option for IBD patients who have previously been treated with VDZ. These insights provide crucial guidance for clinicians when faced with IBD patients who have failed VDZ treatment.

Our study has several limitations. First, as a single-center, retrospective study, it is inherently subject to potential biases, including clinician preferences and selection bias based on patients’ baseline performance status, comorbidities, and individual clinical profiles. These factors may have influenced the choice of biologic therapy and, consequently, the observed outcomes. Second, our study evaluated treatment persistence only over a 52-week period. While persistence is a valuable surrogate marker reflecting the willingness to maintain treatment, ongoing efficacy, and the absence of significant adverse effects, it has its limitations. Persistence does not provide direct objective measures, such as clinical or endoscopic remission, and it may be influenced by non-medical factors like patient adherence or healthcare accessibility. Third, we recognize that the lack of detailed data on anti-TNF serum levels, antibodies, and criteria for dose escalation represents a limitation of our study. This gap may have affected the interpretation of persistence and treatment efficacy outcomes in the anti-TNF group. Lastly, longer-term follow-up studies are necessary to fully assess the sustained efficacy and safety profiles of the different advanced therapies.

## Conclusion

UST demonstrated superior 52-week treatment persistence in IBD patients who had previously been treated with VDZ, compared to those treated with anti-TNF agents, suggesting that UST may be a more favorable option for IBD patients who have failed VDZ treatment. However, this benefit was associated with a need for more frequent dose adjustments.

## Data Availability

The datasets used and/or analysed during the current study are available from the corresponding author on reasonable request.
